# Spaceflight and hind limb unloading induces an arthritic phenotype in knee articular cartilage and menisci of rodents

**DOI:** 10.1038/s41598-021-90010-2

**Published:** 2021-05-18

**Authors:** Andy T. Kwok, Nequesha S. Mohamed, Johannes F. Plate, Raghunatha R. Yammani, Samuel Rosas, Ted A. Bateman, Eric Livingston, Joseph E. Moore, Bethany A. Kerr, Jingyun Lee, Cristina M. Furdui, Li Tan, Mary L. Bouxsein, Virginia L. Ferguson, Louis S. Stodieck, David C. Zawieja, Michael D. Delp, Xiao W. Mao, Jeffrey S. Willey

**Affiliations:** 1 Department of Radiation Oncology, Wake Forest School of Medicine, Winston-Salem, NC 27157 USA; 2 Department of Orthopaedic Surgery, Wake Forest School of Medicine, Winston-Salem, NC USA; 3 Department of Internal Medicine, Section of Molecular Medicine, Wake Forest School of Medicine, Winston-Salem, NC USA; 4 Department of Biomedical Engineering, University of North Carolina, Chapel Hill, NC USA; 5 Department of Cancer Biology, Wake Forest School of Medicine, Winston-Salem, NC USA; 6 Proteomics and Metabolomics Shared Resource, Comprehensive Cancer Center, Wake Forest School of Medicine, Winston-Salem, NC USA; 7 Department of Orthopedic Surgery, Beth Israel Deaconess Medical Center, Harvard Medical School, Boston, MA USA; 8 Department of Mechanical Engineering, University of Colorado At Boulder, Boulder, CO USA; 9 BioServe Space Technologies, Aerospace Engineering Sciences, University of Colorado At Boulder, Boulder, CO USA; 10 Department of Medical Physiology, Texas A&M University Medical School, Bryan, TX USA; 11 Department of Nutrition, Food and Exercise Sciences, Florida State University, Tallahassee, FL USA; 12 Division of Biomedical Engineering Sciences (BMES), Department of Basic Sciences, Loma Linda University, Loma Linda, CA USA

**Keywords:** Physiology, Structural biology, Anatomy, Pathogenesis

## Abstract

Reduced knee weight-bearing from prescription or sedentary lifestyles are associated with cartilage degradation; effects on the meniscus are unclear. Rodents exposed to spaceflight or hind limb unloading (HLU) represent unique opportunities to evaluate this question. This study evaluated arthritic changes in the medial knee compartment that bears the highest loads across the knee after actual and simulated spaceflight, and recovery with subsequent full weight-bearing. Cartilage and meniscal degradation in mice were measured via microCT, histology, and proteomics and/or biochemically after: (1) ~ 35 days on the International Space Station (ISS); (2) 13-days aboard the Space Shuttle Atlantis; or (3) 30 days of HLU, followed by a 49-day weight-bearing readaptation with/without exercise. Cartilage degradation post-ISS and HLU occurred at similar spatial locations, the tibial-femoral cartilage-cartilage contact point, with meniscal volume decline. Cartilage and meniscal glycosaminoglycan content were decreased in unloaded mice, with elevated catabolic enzymes (e.g., matrix metalloproteinases), and elevated oxidative stress and catabolic molecular pathway responses in menisci. After the 13-day Shuttle flight, meniscal degradation was observed. During readaptation, recovery of cartilage volume and thickness occurred with exercise. Reduced weight-bearing from either spaceflight or HLU induced an arthritic phenotype in cartilage and menisci, and exercise promoted recovery.

## Introduction

Osteoarthritis (OA) can lead to pain and disability resulting in decreased health-related quality of life, with associated loss of productivity and high treatment-related costs^1,2^. The reasons for knee OA are thought to be a complex interaction between patient risk factors (age, sex, race, genetic factors, obesity, diet, exercise) and abnormal mechanical loading of the joint (posttraumatic or malalignment)^3^. In particular, articular cartilage and menisci are sensitive to different loading conditions, and the meniscal degradation and loss of function that occurs in relation to risk factors may result in cartilage degradation and subsequent OA^4,5^. While excessive loading from patient obesity^3^ or repetitive loading from high impact activities can contribute to OA development^6,7^, reduced weight-bearing both prescribed and due to a sedentary lifestyle without physical activity have also been associated with cartilage degradation^8–15^.

Reduced weight-bearing studies have demonstrated deterioration of articular cartilage occurs similarly to excessive loading, resulting in cartilage degradation^5,6,10,13–15^. Cartilage degradation, thinning, and loss of glycosaminoglycans (GAGS), which impart compressive properties to cartilage, have been measured in humans after post-surgical limb immobilization and spinal cord injury simulations^10,11,15^. Even partial loading leads to substantial cartilage loss, with 6–8% of cartilage thinning occurring after less than 2 months^10,11^. Reduced weight-bearing in rodent models of hind limb unloading (HLU) via tail suspension, or spinal cord injury, likewise results in cartilage degradation^16–19^. Short-term periods of HLU (13 days) in rats increases pro-arthritic biomarkers in knee cartilage, including matrix metalloproteinases (MMP), and loss of GAG content^19^. These responses are similar to elevated MMP-3 expression and lower GAG content in rat knees after 21 days of limb immobilization^20^. Longer periods of HLU (30 days) in female mice results in articular cartilage loss at the weight-bearing tibial-femoral cartilage contact point, increases MMP-13 concentration with associated decrease in collagen concentration^16^, and alters signaling pathways associated with joint degradation in femoral head articular cartilage^16^. Short-term exposure of chondrocytes to low weight-bearing conditions alters matrix metabolism, however collagen II and proteoglycan production is increased while collagen X is lowered, which could be considered a favorable metabolic response^21^. In general, short and long term periods of reduced weight-bearing alter chondrocyte metabolism and cartilage health, and is disadvantageous to joint health.

Rodents exposed to microgravity in spaceflight experience reduced weight-bearing across joints without the full knee joint immobilization typical of preclinical studies^22^, representing a unique model to study joint tissue responses to unloading. Rapid bone loss and muscle wasting in both rodents and astronauts are well-documented consequences of the skeletal disuse that occurs during periods of microgravity^23,24^. Limited data exist regarding rodent joint responses to spaceflight. Of note, sulfated GAG reduction measured histologically from mouse knee articular cartilage was reported after 30 days in orbit aboard the satellite Bion^25^. Additionally, very few studies have examined the response of menisci to reduced loading in general. The meniscal response to reduced weight-bearing is unclear; preclinical studies identify both degenerative^8,26^ and protective^27,28^ effects of immobilization, but molecular mechanisms for either response are unknown. Further evaluation of microgravity on meniscus cells is necessary to understand the joint degradation that may occur during spaceflight, and the complex relationship these structures play in maintaining joint homeostasis.

The purpose of this study was to evaluate how actual and simulated spaceflight conditions with reduced weight-bearing affect both the cartilage and the meniscus of the medial knee compartment, the most common site of knee OA^29^ that experiences the highest loads across the joint^30^. We hypothesized that reduced-weight bearing would lead to degradation of these structures and a generalized arthritic response. Cartilage and meniscal degradation were measured in male mice after ~ 35 days in orbit aboard the International Space Station (ISS) as part of the Rodent Research 9 (RR9) mission^31^. As a comparative study for the ISS investigation, we examined gross phenotypic and histologic changes in cartilage and menisci from age-matched male mice that were exposed to 30 days of reduced weight-bearing via HLU, followed by a 49 day readaptation (return to weight-bearing) portion that examined if running or climbing exercises could recover any cartilage and meniscal degradation that occurred from 30 days of HLU. The ability of articular cartilage to recover upon a return to normal ambulation and weight-bearing is unclear, with conflicting reports^18,19^. Finally, meniscal responses in female mice were examined in a short-term mission of 13 days in orbit aboard the Space Shuttle Atlantis (STS-135)^32^. The findings of the current study will provide a characterization and quantification of the anatomic and molecular response of multiple knee soft tissues to reduced weight-bearing conditions, including microgravity. In combination with previous preclinical studies, these data further will elucidate how reduced weight-bearing serves as a hazard for whole knee joint health.

## Results

### Thinning and volumetric decline of articular cartilage after spaceflight aboard ISS (RR9)

**Figure 1:**
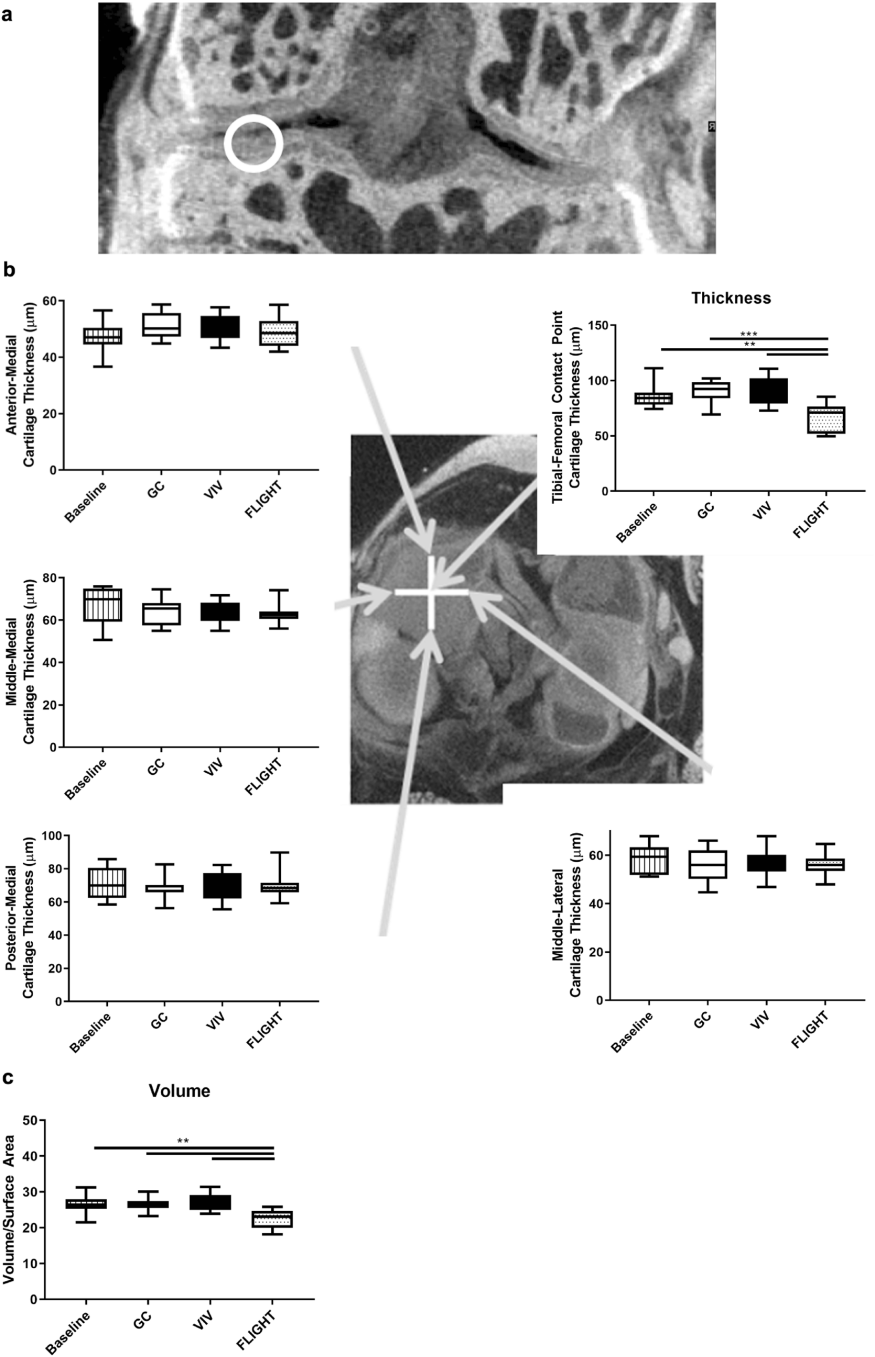
Decreased articular cartilage thickness at the tibial-femoral cartilage-cartilage contact point (indicated at approximately the white circle in the coronal view, and center of the white cross in the axial view, and at the end of the arrow from the upper right plot) and volume lining the medial tibial plateau occurred after spaceflight (FLIGHT) vs Ground Control (GC), Vivarium (VIV), and Baseline. (**a**) Coronal image indicating the contact point. (**b**) Cartilage thickness was lower from FLIGHT compared to controls only at the tibial-femoral cartilage contact point (upper right), but not other regions (n = 10/group). (**c**) Cartilage volume was lower after FLIGHT compared to controls (n = 10/group). **p < 0.01, ***p < 0.001.

Morphometric analyses of the cartilage lining the medial tibial plateau and the medial meniscus from the right knees using contrast enhanced microCT reconstructions^16^ revealed that cartilage thinning occurred specifically at the weight-bearing tibial-femoral cartilage contact point vs controls after ~ 35 days in orbit in FLIGHT vs. ground controls (GC) and vivarium controls (VIV) (− 25.4% vs GC, *p* = 0.00070; − 24.7% vs VIV, *p* = 0.0011) (Fig. 1a,b). Thinning was not observed at other regions throughout the tibial plateau. The volume of the articular cartilage was lower after FLIGHT (− 15.4% vs GC, *p* = 0.0033; − 15.8% vs VIV; *p* = 0.0024) (Fig. 1c). Importantly, no differences were observed in cartilage thickness or volume between the baseline control group or either GC or VIV (Fig. 1b,c); however thickness (− 21.6%, *p* = 0.0072) and volume (− 15.4%, *p* = 0.0034) in FLIGHT mice was significantly lower than baseline.

### Volumetric decline and reduced sulfated GAG content of the menisci after spaceflight aboard ISS (RR9)

**Figure 2:**
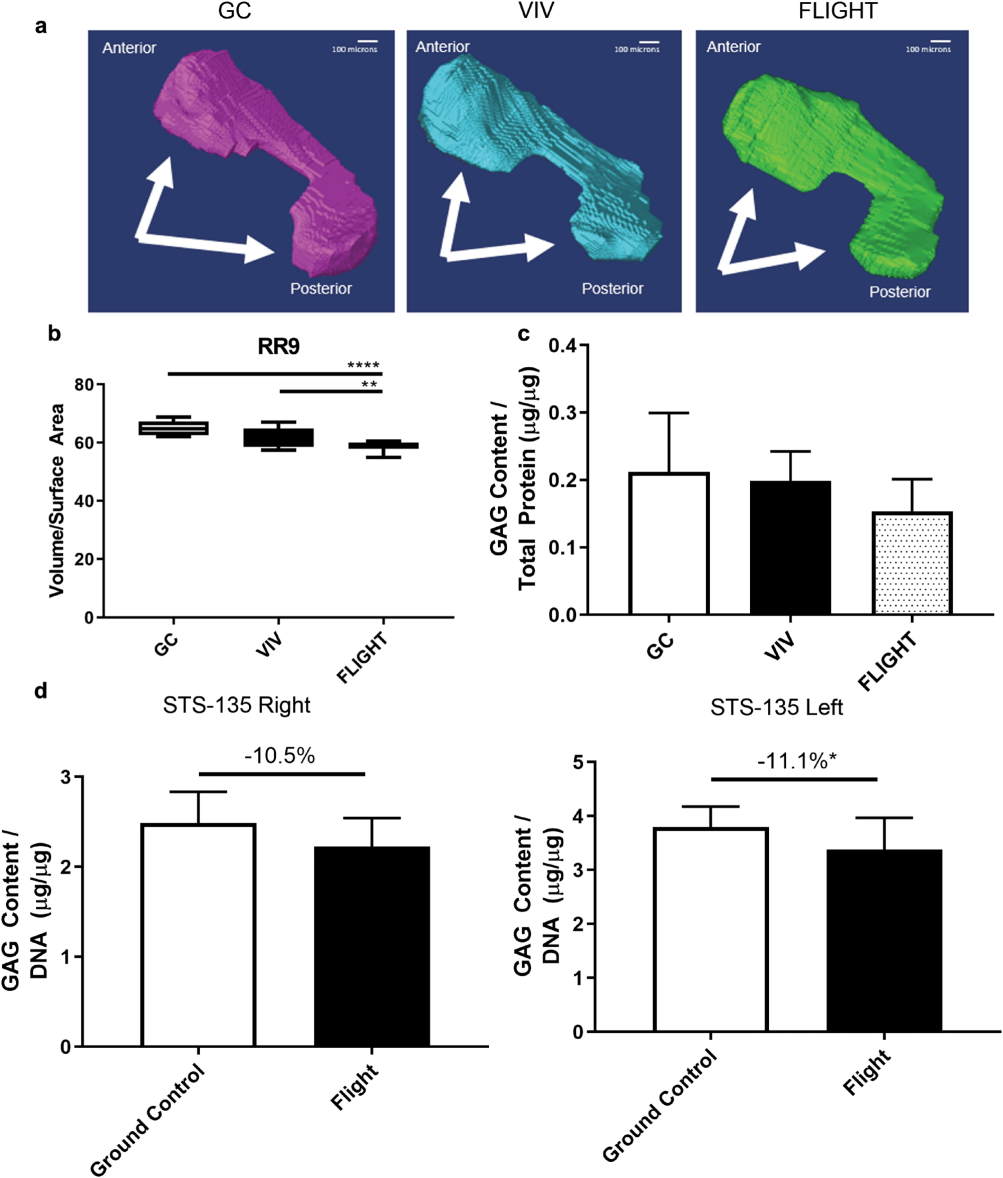
Meniscal volume and sulfated glycosaminoglycan (GAG) content is reduced after spaceflight. (**a**) Representative 3D reconstructions of medial menisci used for biometric analysis, with arrows identifying the respective anterior and posterior horns. (**b**) Meniscal volume measurements were lower in FLIGHT compared to controls (n = 10/group). (**c**) GAG content determined via a dimethylmethylene blue assay was not significantly lower in RR9 FLIGHT vs controls (n = 10/group). (**d**) GAG content from menisci harvested from the right and left knees (side identified in each plot) of mice flown on STS-135 were significantly (Left, n = 12–14/group) or marginally (Right, n = 8/group) lower than Ground Control. *p < 0.05, **p < 0.01, ****p < 0.0001. Scale bar = 100 µm.

Medial meniscal volume (Fig. 2a) from FLIGHT was lower versus controls (− 9.5% vs GC, *p* < 0.00010; − 5.9% vs VIV, *p* = 0.0095) (Fig. 2b). Sulfated GAG content was lower after FLIGHT vs GC, though not significant (− 27.7% vs GC, *p* = 0.31; − 22.8% vs VIV, *p* = 0.77) (Fig. 2c).

### Reduced sulfated GAG content in the menisci after spaceflight aboard the Space Shuttle Atlantis (STS-135)

Similar to the response observed in long-duration flight, sulfated GAG content in the pooled medial and lateral menisci after ~ 13 days of spaceflight as part of STS-135 was significantly lower vs GROUND control in the left knee (− 11.1% vs GC, *p* = 0.042), and marginally lower than GROUND control in the right knee (− 10.5% vs GC, *p* = 0.14) (Fig. 2d). Notably GAG content decreased even though these mice were growing throughout the 13 day experiment.

### Reduced matrix components and increased metalloproteinases within cartilage and menisci after spaceflight aboard ISS (RR9)

**Figure 3:**
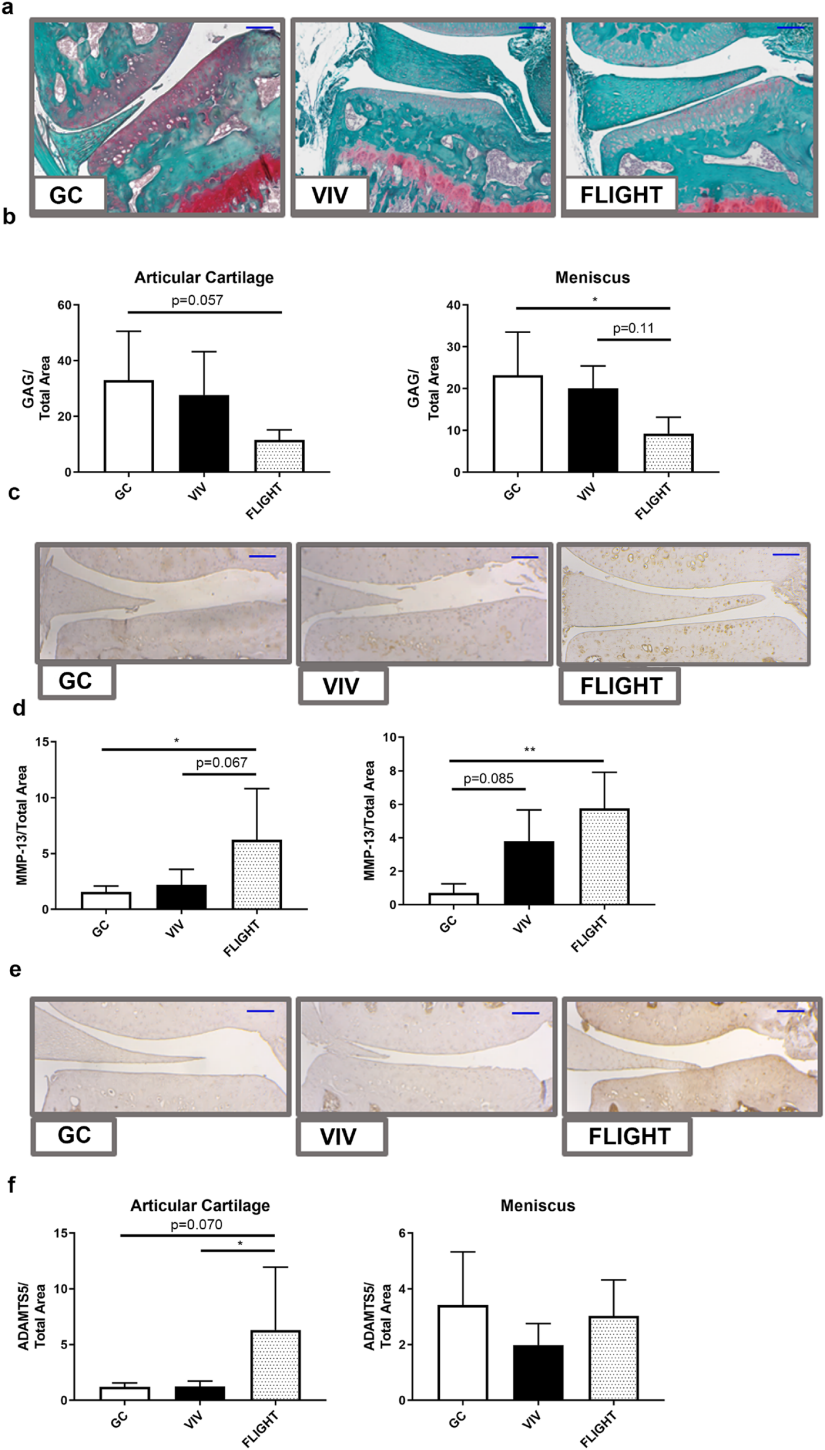
Sulfated glycosaminoglycans (GAG) are lower and catabolic enzymes are higher within the articular cartilage lining the medial tibial plateau and the medial meniscus from mice after spaceflight (FLIGHT) vs Ground Control (GC) and Vivarium Control (VIV). (**a**) Representative safranin-O stained sections (n = 5–7/group). (**b**) Sulfated GAGs in the articular cartilage are lower in FLIGHT vs GC, and vs GC and VIV in meniscus (n = 5–7/group). (**c**) Representative MMP-13 stained sections (n = 5–7/group). (**d**) MMP-13 staining was higher in the articular cartilage and menisci in FLIGHT vs GC and VIV (n = 5–7/group). (**e**) Representative ADAMTS5 stained sections (n = 5–7/group). (**f**) ADAMTS5 was higher in FLIGHT vs both GC and VIV in articular cartilage (n = 5–7/group). *p < 0.05, **p < 0.01. Scale bar = 100 µm.

Sulfated GAGs measured histochemically (Fig. 3a) using safranin-O staining were reduced within the articular cartilage lining the medial tibial plateau (− 65.0% vs GC, *p* = 0.057) and the medial meniscus (− 60.2% vs GC, *p* = 0.023; − 53.8% vs VIV, *p* = 0.11) (Fig. 3b). The expression of the type II collagen-degrading MMP-13 (Fig. 3c) was elevated throughout the articular cartilage lining the medial tibial plateau of FLIGHT mice vs controls (+ 298% vs GC; *p* = 0.031, + 184% vs VIV, *p* = 0.067) (Fig. 3d) Likewise, MMP-13 was elevated in menisci from FLIGHT mice vs GC (712%, *p* = 0.0064). The proteoglycan-degrading ADAMTS5 (Fig. 3e) expression within the articular cartilage lining the tibial plateau was greater after FLIGHT vs controls (+ 423% vs GC, *p* = 0.070; + 406% vs VIV, *p* = 0.045) (Fig. 3f). OARSI scores that indicate the severity of OA, measured from histologic sections, were similar between all groups [mean(SD)]: [GC 0.19(0.26SD); VIV 0.39(0.42); and FLIGHT 0.22(0.36)].

### Proteomic analysis of menisci after spaceflight characterize arthritic responses (RR9)

**Figure 4:**
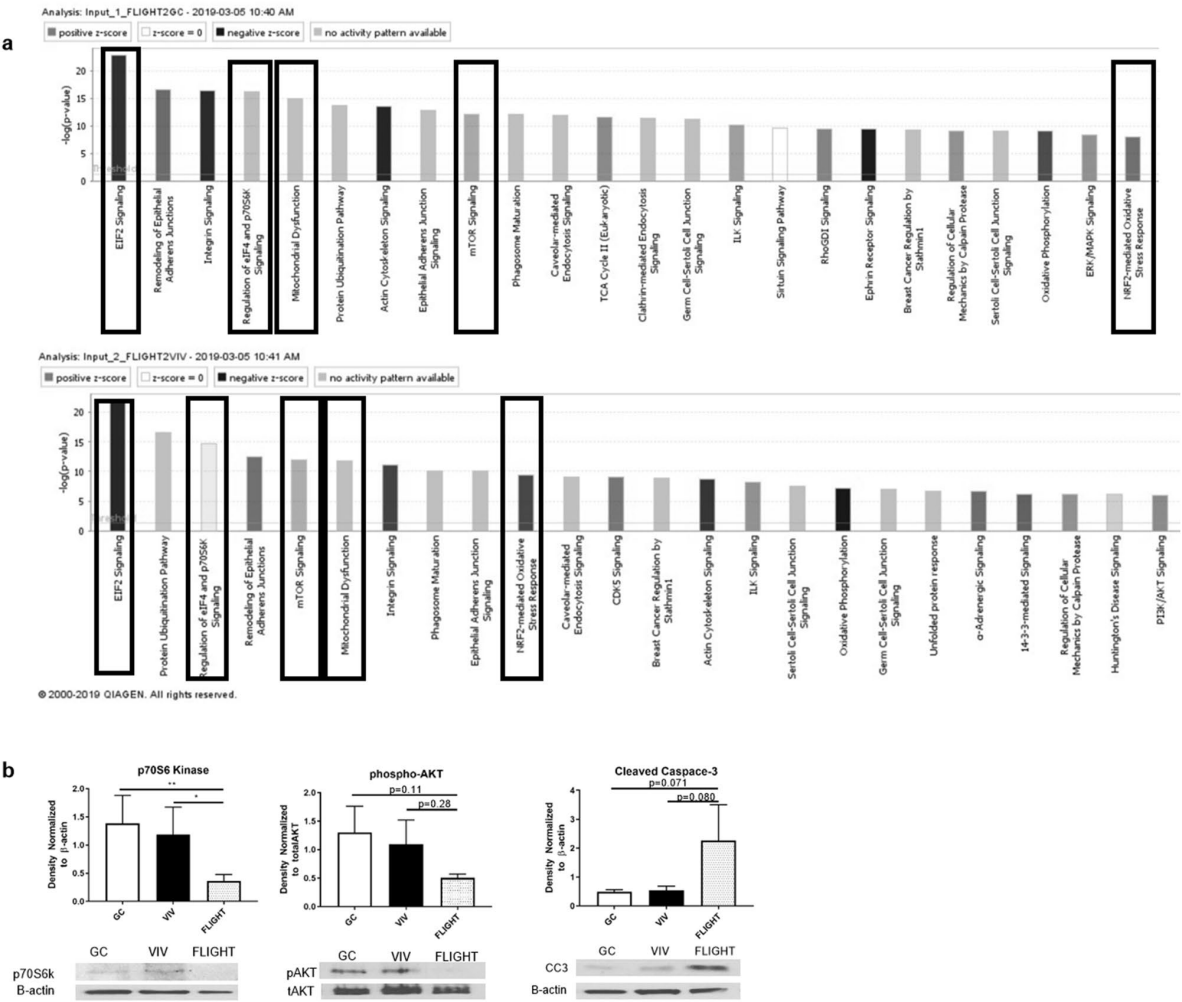
Ingenuity Pathway Analysis (IPA) identifying altered canonical pathways, and supportive Western blot analyses (**a**) Canonical pathways comparisons of FLIGHT vs GC (top) and FLIGHT vs VIV (bottom) indicate involvement of catabolic response and increased oxidative stress via p70S6K, mitochondrial dysfunction, mTOR signaling and NRF2-mediated oxidative stress; color coding indicates directional change in pathway activity (n = 10/group). (**b**) p70S6 Kinase was reduced vs controls post-FLIGHT (n = 3/group); pAKT (s473) was marginally lower post flight (n = 3/group); Cleaved Caspase-3 was increased post-flight (n = 3/group). *p < 0.05, **p < 0.01.

Mass spectroscopy identified 292 significantly altered proteins out of 2129 total proteins when comparing FLIGHT menisci to either GC or VIV. These 292 significant proteins were imported into an Ingenuity Pathway Analysis (IPA) software to detect altered pathways due to spaceflight by comparing between FLIGHT vs GC, FLIGHT vs VIV, and GC vs VIV. Hierarchical clustering of all canonical pathways detected significant responses of signaling pathways in menisci of FLIGHT vs both control sets (Supplemental Fig. 1). Of those pathways, 4 of the top cluster and NRF2 mediated oxidative stress were selected for further assessment and further validation via Western blotting, due to shared differences in protein abundance for GC and VIV vs FLIGHT (Table 1 and Fig. 4a), and known associations with an arthritic molecular phenotype and due to the described histologic and GAG results. These pathways included: mitochondrial dysfunction; altered EIF2 signaling; reduced NRF2 signaling (oxidative stress); lowered eIF4/p70S6K signaling; and lowered mTOR signaling (Fig. 4a). IPA assigned proteins with a log2 fold change (an equivalent expression log ratio) > 1.5 (+ /-) and had a *p* value < 0.05 were identified to each canonical pathway (Fig. 4a) as follows: 31 proteins in the EIF2 pathway; 21 proteins in mitochondrial dysfunction pathway; 22 proteins in NRF2-mediated oxidative stress response; 24 proteins in regulation of eIF4 and p70S6K signaling; and 26 proteins in mTOR pathways were significantly different in FLIGHT vs both GC and VIV (Table 1).

**Table 1:** Shared proteins in the menisci of ground control and vivarium control vs flight mice that are clustered within differential canonical pathways between both controls and flight determined via ingenuity pathway analysis.

EIF2	Mitochondrial dysfunction	NRF2	P70S6K	mTOR
EIF2S2	VDAC1	GSTP1	RPS5	Cdc42
EIF2S3	ATP6F1A	RAP1A	RAP1A	EIF3B
EIF3B	ATP5PD	RAP1B	PPP2R1B	EIF3E
EIF3E	GPX4	EPHX1	RAP1B	EIF3G
EIF3G	ATP5MX	VCP	EIF2S3	EIF4A1
EIF4A1	NDUFV1	MGST3	RPS21	EIF4A2
EIF4A2	ATP5F1C	MAP2K1	MAP2KA	FKBP1A
HRAS	OGDH	GSTM3	RPS16	HRAS
HSPA5	SDHA	PRDX1	RPS4Y1	PPP2R1A
MAP2K1	FIS1	GSTM5	PPP2R1A	PPP2R1B
PABPC1	CYB5A	SOD3	RPS9	PPP2R2A
PPP1CA	HSD17B10	MGST1	RPS20	RAP1A
PPP1CB	UQCRC2	DNAJB11	RPS3	RAP1B
RPL13A	PARK7	GSTK1	EIF4A1	RHOA
RPL18A	ACO1	DNAJA2	RPS24	RHOC
RPL22	NDUFA9	RRAS	EIF2S2	RHOG
RPL24	ATP5PB	CCT7	RRAS	RPS2
RPL4	AIFM1	DNAJA1	PPP2R2A	RPS3
RPL5	NDUFA11	HRAS	HRAS	RPS5
RPL6	ACO2	QO2	EIF3E	RPS9
RPL7	NDUFA8	PPIB	PABPC1	RPS16
RPL7A		DNAJB4	EIF4A2	RPS20
RPL9			RPS2	RPS21
RPS16			EIF3G	RPS24
RPS2				RPS4Y1
RPS20				RRAS
RPS24				
RPS3				
RPS4Y1				
RPS9				
RRAS				

Listed proteins are significantly different in both GC and VIV vs FLIGHT with a fold change of 1.5 or greater (*p* < 0.05).

### Validation of proteomic assessment identifying arthritic responses via western blotting

Western blotting was used to partially validate mass spectroscopy and IPA data (Supplemental Fig. 2). P70S6K, a downstream molecule of mTOR and AKT pathway was significantly decreased in FLIGHT group when compared to both GC and VIV (− 74.2% vs GC, *p* = 0.0053; − 69.9% vs VIV, *p* = 0.022) (Fig. 4b), while pAKT (s473) concentration was marginally lower after FLIGHT (− 61.1% vs GC, *p* = 0.11; − 53.9% vs VIV, *p* = 0.28) (Fig. 4b), all of which are aligned with alterations in mTOR/AKT signaling pathways. The expression of cleaved caspase-3, a marker for apoptosis was marginally greater after FLIGHT (+ 363% vs GC, *p* = 0.071; + 318% vs VIV, *p* = 0.080) (Fig. 4b).

### Thinning and volumetric decline of articular cartilage and medial menisci after 30 days of HLU

**Figure 5:**
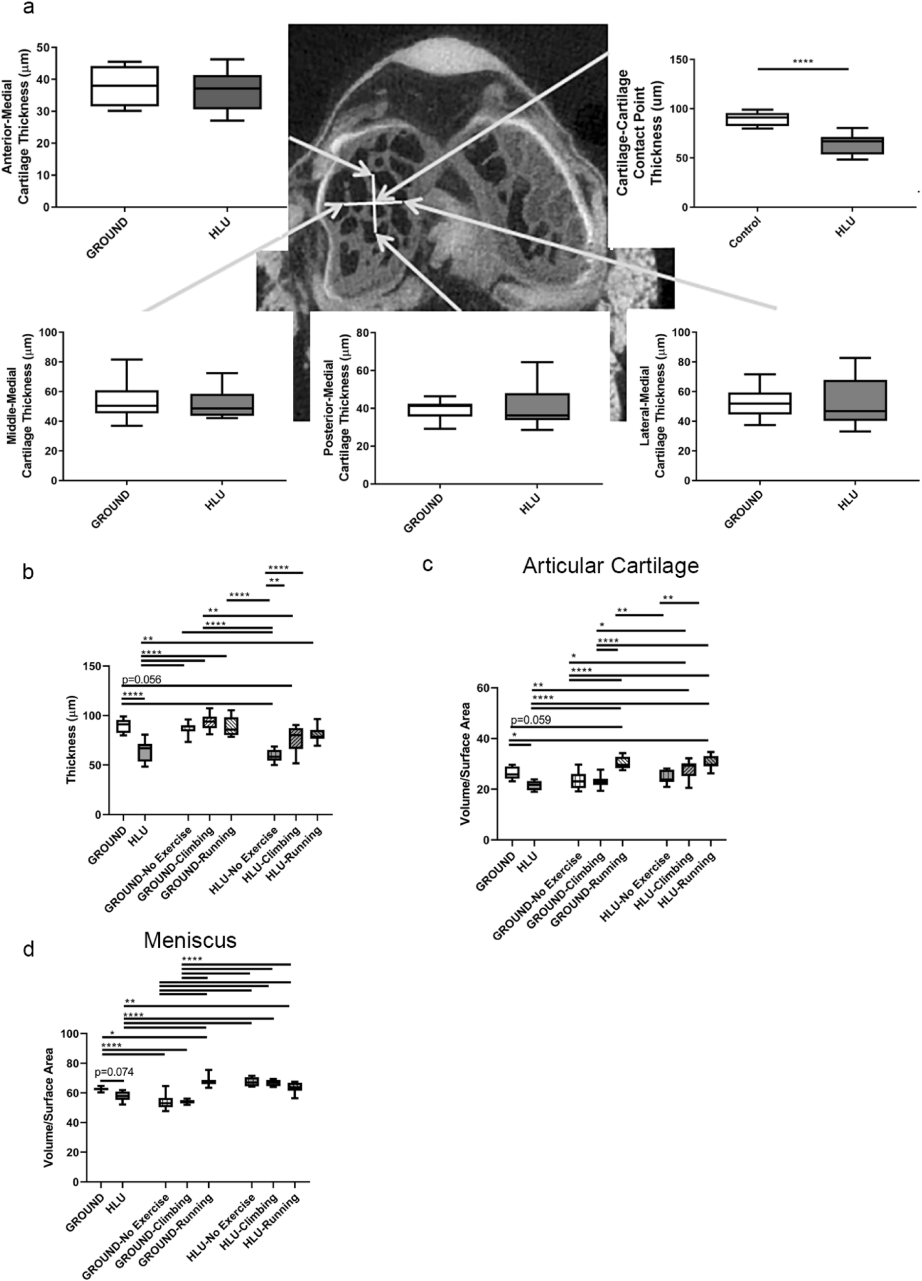
Exercise after a 30 day period of HLU results in recovery of cartilage and meniscal thickness and volume (n = 7–10/group). Arrows point to the approximate location of where measures were performed using 3D reconstructions of cartilage overlying the bone (**a**) Cartilage thickness at the tibial-femoral cartilage-cartilage contact point was lower in HLU compared to GROUND after 30 days, but not at other regions of the tibial plateau. (**b**) Thinning of cartilage at the tibial-femoral cartilage contact point remained in mice that had previously been HLU but returned to full weight-bearing from Days 31–80 but performed No Exercise. However, thickness of cartilage at the tibial-femoral contact point was significantly greater in previously HLU mice with both Climbing and Running from Days 31–80 than HLU-No Exercise. (**c**) Loss of cartilage volume observed throughout the medial tibial plateau was observed after Day 30 of HLU. For the mice that were previously HLU, recovery of cartilage volume was observed in HLU-Climbing and HLU-Running by Day 80. (**d**) Meniscal volume was marginally lower in HLU vs GROUND on DAY 30. Readaptation to full weight bearing from Days 31–80 in all HLU groups (HLU-No exercise, HLU-Climbing, and HLU-Running) resulted in recovery vs HLU on Day 30. *p < 0.05, **p < 0.01, ****p < 0.0001.

Cartilage thinning occurred specifically at the weight-bearing tibial-femoral cartilage contact point vs controls after ~ 30 days of HLU vs GROUND (− 28.7%, *p* < 0.00010) (Fig. 5a,b). Thinning was not observed at other regions throughout the tibial plateau (Fig. 5a). The volume of the articular cartilage was also lower after HLU (− 17.8%, *p* = 0.017) (Fig. 5c). The volume of the medial meniscus was marginally lower after HLU (− 7.8%, *p* = 0.074) (Fig. 5d).

### Reduced matrix components and increased metalloproteinases within cartilage and menisci after HLU

**Figure 6:**
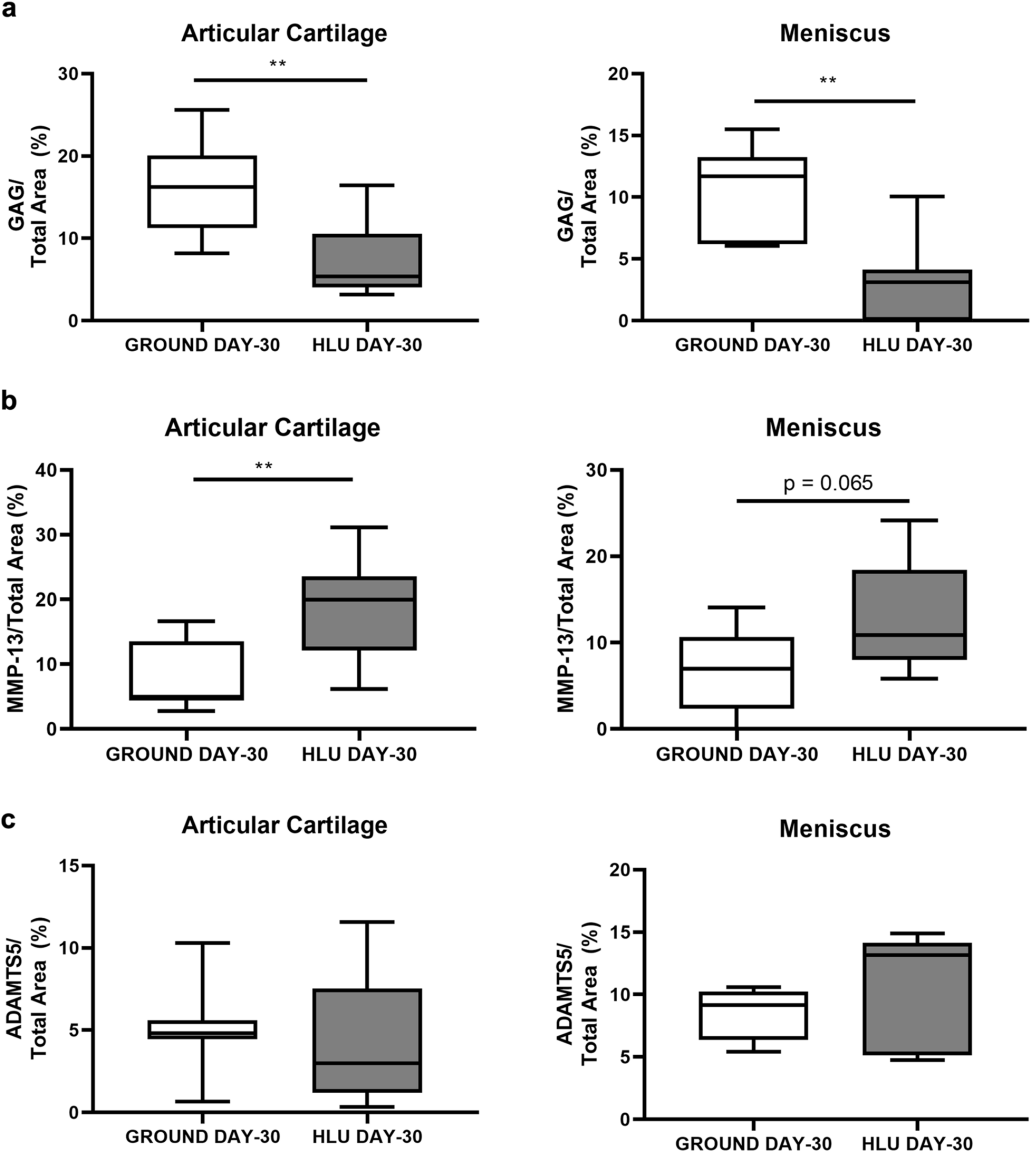
Sulfated glycosaminoglycan (GAG) abundance was lower and catabolic enzymes elevated within the articular cartilage lining the medial tibial plateau and the medial menisci from mice after HLU. (**a**) Sulfated GAG abundance was lower in both the articular cartilage and menisci after 30 Days of HLU (n = 7–8/group). (**b**) MMP-13 was significantly increased in the articular cartilage after HLU, and was marginally greater in the meniscus (n = 7–8/group). (**c**) ADAMTS5 was similar between both GROUND and HLU on Day 30 (n = 7–8/group). **p < 0.01.

Sulfated GAGs measured histochemically by safranin-O staining (Fig. 6a) were reduced within both the articular cartilage lining the medial tibial plateau (− 55.4%, *p* = 0.0020) and the medial meniscus (− 71.7%, *p* = 0.0016) from HLU DAY-30 vs GROUND DAY-30. The expression of the type II collagen-degrading MMP-13 staining was elevated throughout the medial tibial plateau of HLU DAY-30 vs GROUND DAY-30 (+ 122%, *p* = 0.0047) and the medial meniscus (+ 85.0%, *p* = 0.065) (Fig. 6b). The expression of ADAMTS5 did not change after HLU compared to GROUND (Fig. 6c). OARSI scores were not significantly different between groups [mean(SD)]: [GROUND 0.33(0.43), and HLU 0.50(0.76)].

### Recovery of articular cartilage after ground-based HLU is dependent on exercise

Cartilage Thickness: As noted, by Day 30, cartilage thickness measured at the tibial-femoral contact point was significantly lower in HLU mice than GROUND controls (− 28.7%, *p* < 0.00010) (Fig. 5a). In the GROUND mice that subsequently underwent exercise for Days 31–80, exercise did not affect thickness of cartilage at the tibial-femoral cartilage contact point or promote growth (Fig. 5b). However, in mice that had been previously HLU but were not exercised (HLU-No Exercise), the significant thinning of cartilage remained on Day 80 relative to GROUND mice on Day 30 (− 34.3%%, *p* < 0.00010) (Fig. 5b), and also vs GROUND-No Exercise mice on Day 80 (− 32.2%; *p* < 0.00010) (Fig. 5b). Moreover, marginal cartilage thinning remained in the mice that had been previously HLU but performed climbing exercise (HLU-Climbing) relative to GROUND on Day 30 (− 15.1%, *p* = 0.056). In contrast, running exercise 3X weekly significantly recovered cartilage thickness by Day 80 in mice that had previously been HLU (HLU-Running) vs HLU measured on Day 30 (+ 25.8% vs HLU, *p* = 0.0052), and was significantly thicker than the HLU-No Exercise mice on Day 80 (+ 36.6% vs HLU-No Exercise, *p* < 0.00010) (Fig. 5b). Likewise, cartilage thickness in HLU-Climbing mice on Day 80 was greater than HLU-No Exercise (+ 29.3%, *p* = 0.0044) (Fig. 5b).

Cartilage Volume: As noted, by Day 30, cartilage volume was significantly lower in HLU mice than GROUND (− 17.8%, *p* = 0.022) (Fig. 5c). After 49 days, GROUND-Running mice had greater volume on Day 80 vs. HLU on Day 30 (+ 40.8% vs HLU, *p* < 0.00010), and was also greater vs GROUND-No Exercise and GROUND-Climbing measured on Day 80 (+ 30.0% vs GROUND-No Exercise, *p* < 0.00010; + 31.6% vs GROUND-Climbing, *p* < 0.00010) (Fig. 5c). Moreover, cartilage volume from GROUND-Running mice on Day 80 was marginally greater than GROUND measured on Day 30 (+ 15.7%, *p* = 0.059). For the mice that were previously HLU, recovery of cartilage volume was observed in HLU-Climbing vs HLU on Day 30 (+ 29.8%, *p* = 0.0010) , and also in from HLU-Running on Day 80 vs HLU on Day 30 (+ 41.5, *p* < 0.00010) (Fig. 5c). The volume of cartilage was greater on Day 80 in HLU-Running vs HLU-No Exercise (+ 23.5%; *p* = 0.0012). Finally, the volume of cartilage was greater on Day 80 in HLU-Running vs GROUND measured on Day 30 (+ 16.3%; *p* = 0.040).

Meniscal Volume: Meniscal volume was marginally lower by Day 30 after HLU (− 7.8%, *p* = 0.074) (Fig. 5d). Interestingly, meniscal volume was lower for GROUND-No Exercise and GROUND-Climbing mice at Day 80 relative to GROUND Day 30 (− 13.4% vs GROUND-No Exercise, *p* < 0.00010; − 13.5% vs GROUND-Climbing, *p* < 0.00010) (Fig. 5d). Meanwhile, GROUND-Running mice exhibited significantly greater meniscal volume when compared to both GROUND and HLU mice on Day 30 (+ 8.3% vs GROUND, *p* = 0.040; + 17.4% vs HLU, *p* ≤ 0.00010), and also greater volume than all GROUND animals on Day 80 regardless of exercise (+ 25.1% vs GROUND-No Exercise, *p* < 0.00010; + 25.2% vs GROUND-Climbing, *p* < 0.00010) (Fig. 5d). Meniscal volume from all groups of mice that were previously HLU was significantly greater than the volume measured from HLU mice on Day 30, including HLU-No Exercise (+ 16.5% vs HLU, *p* ≤ 0.00010), HLU-Climbing (+ 15.6% vs HLU *p* ≤ 0.00010) and HLU-Running (+ 10.4%, *p* = 0.0097) (Fig. 5d).

## Discussion

Reduced weight-bearing prescribed as treatment for orthopaedic conditions or as part of a sedentary lifestyle can lead to joint impairment and degradation^8–15^. Rodent spaceflight studies examining skeletal responses both on-orbit and using ground-based analogues (e.g., HLU) are good models to characterize the effects on joint health. While the response to spaceflight and reduced weight-bearing on bone and muscle have been well-documented^16,33–35^, the effects on articular cartilage and menisci during spaceflight are poorly understood despite the relevance for astronaut health and performance, and as an analogue for reduced weight-bearing on the ground^36,37^. In this study, both the articular cartilage lining the medial tibial plateau and stabilizing menisci within knees developed arthritic responses to periods of reduced weight-bearing during spaceflight and from HLU on Earth. The most important findings of this study are that: 1] cartilage degradation after both spaceflight for 35 days and HLU for 30 days occurs at the point of greatest tibial-femoral cartilage-cartilage contact during weight-bearing in both conditions. As no differences were seen between Baseline and other controls with regard to thickness or volumetric changes, the differences post-flight represent change vs reduced growth; 2] cartilage and meniscal degradation from HLU in male mice resembles the extent and spatial distribution of degradation that has previously been observed to occur in female mice^16^ and male rats^19^; and, 3] recovery of cartilage volume and thickness is possible with exercise upon a return to joint loading. In the context of these findings, it is important to identify that rodents that are part of the rodent flight studies are active in-flight, and have been shown to locomote primarily with forelimbs and thus experienced highly reduced (but not abolished) weight-bearing across hind limb joints^22^.

With regards to astronaut health and performance, maintenance of skeletal health is a critical component of mission success. Normal loads applied across synovial joints maintain health and performance, particularly with regards to the knee^38,39^. Thus, reduced weight-bearing experienced during spaceflight represents a potential challenge to synovial joint health^4,8,10–15,19,37^. The cartilage degradation observed in mice during HLU and spaceflight is consistent with the limited number of existing rodent studies examining cartilage health from simulated spaceflight conditions^16,17,19^ and in agreement with the proteoglycan loss that occurred in rodents during a period of spaceflight aboard Bion^25^. The loss of cartilage volume and thickness in both HLU and FLIGHT knees occurred with elevated matrix metalloproteinase concentration in both the articular cartilage and menisci. Several molecular effectors of OA were observed within the menisci^40^. The molecular arthritic phenotype indicating increased oxidative stress in the menisci after spaceflight involved: lower NRF2 signaling coincident with reduced antioxidant enzymes abundance (e.g., superoxide dismutase (SOD3) and glutathione peroxidase (GPX4), Table 1); reduced phosphorylation of Akt and downstream anabolic signaling components (mTOR and p70S6K); lower protein synthesis (decreased EIF2 signaling); and increased MMP-13 expression. These responses may promote the volumetric loss that was observed after both spaceflight and HLU. In addition, IPA analysis also indicated mitochondrial dysfunction, which contributes to cartilage/meniscus cell apoptosis (indicated by increased cleaved caspase 3) and degradation of joints^40,41^. Mitochondrial dysfunction within the menisci after spaceflight is also in agreement with altered mitochondrial dysfunction and stress observed during and after spaceflight from multiple rodent tissues and astronauts^24^ These molecular and phenotypic responses in the menisci are similar in nature to the response observed in the knee and hip articular cartilage from female mice after 30 days of HLU^16^. Similar catabolic mechanisms and responses have also been observed in other in vitro and in vivo osteoarthritic models, with increased oxidative stress, reduced anabolic pathways, and evidence of mitochondrial dysfunction^42–47^. Clinically, circulating MMP levels that are also associated with collagen degradation with arthritis (e.g., MMP-3) were increased during a 21 day head-down tilt bedrest study as a spaceflight microgravity analogue^48^. Interestingly, several of the observed responses (e.g., reduced SOD2) are also characteristic of arthritic responses that occur with an abnormal excess of loading across joints^44^. However, OARSI scoring did not indicate pronounced degradation of the knee joint tissues, in agreement with other flights of similar duration^25^ suggesting early molecular changes occurring in joint tissue before histological onset of osteoarthritic degradation occurs. As such, the degradation and molecular responses in the articular cartilage and menisci could, with progression, predispose astronauts to arthrosis and later symptomatic arthritis resulting from the microgravity environment.

This study considered the microgravity conditions experienced aboard the ISS and space shuttle as analogues for the clinical scenarios of reduced weight-bearing. However, as noted, progressive joint degradation associated with the spaceflight environment would also act as a risk factor to human health and performance during long-duration missions^36^, particularly when occurring with persistent bone damage that could predispose to rapid joint destruction and fracture upon reaching a destination^23,33,34^. Exercise regimens during spaceflight have long been employed as a means to preserve musculoskeletal health by increasing applied loads imparted externally via reaction forces or internally from muscle contractions. Long-term use of exercise modalities such as the Advanced Resistive Exercise Device (ARED) are utilized by US astronauts during stays in orbit aboard ISS as a means to protect bone and muscle health^49^. Use of the ARED alone (without bisphosphonates) has been efficacious at maintaining bone over the course of ISS missions of ~ 6 months^23^, though some deficits remain post-flight vs preflight. However, by 1 year post-flight, deficiencies in bone can exceed the magnitude observed upon return from ISS^23^, indicating bone damage progression occurs with a return to normal gravitational loading despite some preservation in flight. This progressive decline in bone mineral density and estimated strength deficits occurs in astronaut cohorts who performed ARED + bisphosphonate treatment in-flight^23^. This is concerning with regard to bone health upon reaching destinations during a long-duration mission (e.g., trips to Mars), as a fracture of a lower limb element on the planet surface would be mission critical. As Mars provides an uneven and potentially dangerous terrestrial environment, dynamic loads during normal locomotion or rapid egress increase this risk of joint soft tissue failure, particularly if the underlying and surrounding bony tissues are compromised. Progressive degradation of the menisci could result in acute failure during rapid motion, such as during egress or locomotion over the terrain possibly leading to meniscal tears and subsequent disability. Thinned menisci could further potentiate joint damage due to the abnormal shear stresses applied to underlying articular cartilage during such dynamic loading motions^50–53^. Thus, thinned structures that may progress toward symptomatic arthritis, and even in the absence of symptomatic OA as identified histologically with OARSI scoring^16^, may be sufficient to increase the risk of skeletal failure upon reaching a destination or during a rapid transit or egress motion.

A primary clinical concern involving reduced weight-bearing after an orthopaedic injury or from sedentary lifestyle is that the subsequent joint degradation may not be recoverable and may become permanent. Cartilage has limited repair capacity^14^, and consideration must be given regarding the differences between mouse and human articular cartilage repair capacity. Human articular cartilage is divided into multiple zones (superficial, middle, and deep zones). These zones vary in matrix biochemical composition, cell density and morphology. Superficial chondrocytes are known to have proliferative capacity compared to middle and deep zone chondrocytes. Presence of tidemark hinders the vascularization of articular cartilage which restricts its healing/repair process upon injury^54^. Mouse joints are small with thin cartilage, which consists of only a few cell layers and the presence of open growth plates through advancing age likely increase intrinsic healing potential^55^. However, as noted, periods of reduced weight-bearing or HLU have been found to induce cartilage damage in humans, rodents, and dogs^9,13,56,57^. The current study reaffirms those findings, demonstrating the area of greatest cartilage decrease is at the point of greatest tibial-femoral cartilage-cartilage contact. Conversely, it also revealed that specific joint-loading exercises have the potential to reduce and recover cartilage damage after a period of reduced weight-bearing, suggesting these exercises have utility in attenuating arthritic responses in human joints as well. As previously noted, astronauts in spaceflight who perform ARED are able to maintain bone health during spaceflight to lessen the degree of degradation upon return to normal loading^23^, strengthening the association that these tested exercises confer some joint protection and recovery. In clinical patients with weight-bearing restrictions or a sedentary lifestyle, a prescribed exercise routine may be an effective way to preserve joint function and reduce the degradation of the cartilage and menisci. Elucidating the clinical response to specific exercise in a reduced weight-bearing scenario may help identify a possible therapeutic pharmacological target, and requires further research. Based upon the results of the present study, running on flat surfaces may be superior to climbing stairs after a period of reduced loading to assist in joint recovery and mitigating arthritic responses, though there may be other exercises that are better suited to slowing joint degradation that have not yet been tested. The increased contact loads experienced with running may provide such benefit. However, this study exemplifies the similarities in joint degradation between microgravity experienced during spaceflight and clinical reduced weight-bearing conditions on the ground, and highlights the possibility of joint recovery in both scenarios with appropriately prescribed exercise.

This study is not without limitations. The first considers HLU as an analogue for FLIGHT. Both conditions experience reduced weight-bearing on the hind limb, though the process of HLU could result in a greater degree of decreased loading vs FLIGHT, in which mice are active^22^. However, it should be noted that gross loss of cartilage was observed from FLIGHT and HLU mice in similar spatial locations vs each other and relative to multiple controls, and also relative to previous work using older, female mice of the same stain that experienced HLU for 30 days^16^. Thus these models appear appropriate based on the outcomes presented. Our previous study^19^ simulating spaceflight conditions (e.g., low dose radiation + / − reduced weight-bearing via HLU) responses in the knees of rats utilized more advanced imaging modalities (e.g., 7 T(T) MRI) that provided both morphologic changes and insight into incipient molecular deficits, such as collagen network disruption. While contrast-enhanced CT scans utilized for this study provided the ability to spatially localize deficits across the knees of the smaller mouse model and compare results with our previous HLU + / − radiation mouse study^16^, it cannot provide those molecular correlates. Moreover, for the HLU study, tissues were harvested upon removal from HLU, while for flight tissues were harvested ~ 24 h post-recovery. This unavoidable delay in returning live animals could affect gene expression patterns for multiple proteins and affect metabolism in several ways; however, it is unlikely this period of loading would affect the gross morphology of the cartilage and/or the menisci. Future missions may likely return live animals near the Kennedy Space Center, and will reduce recovery time.

In conclusion, reduced weight-bearing from either spaceflight or HLU induced an arthritic phenotype with degradation of knee articular cartilage and menisci in male mice. Cartilage loss occurred specifically at the region of greatest weight-bearing and tibial-femoral cartilage-cartilage contact (and thus presumably the location that experiences the greatest reduction of applied loads in the experimental conditions), which is consistent with findings from female mice after 30 days of HLU^16^. Furthermore, exercise after a period of joint unloading reduced cartilage degradation and improved thickness. As an analogue for reduced weight-bearing in clinical scenarios, these results suggest that prescribed exercise programs may alleviate and recover articular cartilage damage. Exploring the utilization of these exercises for joint recovery in patients with prescribed reduced weight-bearing, who have a sedentary lifestyle, or most directly during and/or after spaceflight and, may lead to a better understanding of the chondro-protective effects of exercise.

## Methods

Three sets of animal studies were performed: two involving spaceflight, and another with a ground-based, reduced weight-bearing microgravity analogue. The study was carried out in compliance with the ARRIVE guidelines.

### Rodent research-9 (RR9)

All experiments were approved by the NASA Ames Institutional Animal Care and Use Committee (IACUC), Kennedy Space Center IACUC, and the Loma Linda Medical Center IACUC, and conformed to the *Guide for the Care and Use of Laboratory Animals* (National Institutes of Health, Bethesda, MD, USA). Male C57BL/6 J mice (Jackson Labs, Bar Harbor, ME) were sent to the ISS as part of the Rodent Research (RR)-9 mission in August 2017, spending ~ 35 days in microgravity. The RR9 experiment was launched as part of the SpaceX Commercial Resupply Services (CRS)-12 mission from the Kennedy Space Center (KSC) at Cape Canaveral, FL to the ISS. Mice arrived 4 weeks prior to launch at the KSC and were maintained on a 12:12 h dark/light cycle, and housed in vivarium caging (n = 10/cage). Mice were skeletally growing at the outset^58^. Prior to launch, six cages of mice were separated into three different groupings, with 2 cages per group. Mouse sample size per group (n = 20) was determined by NASA prior to the flight, in accordance with housing capacity of the Rodent Habitats aboard the ISS. Individual animal randomization was avoided to prevent complications related to potential altered hierarchical structure within cages; instead groups were determined by finding the mean mass of mouse per cage so that each group consisted of cages that each had similar body masses. Nine-week old male mice were separated into the following groups: 1] mice to be launched to the ISS (FLIGHT; n = 20) that are housed on-orbit within specialized Rodent Habitats that provide food and water to mice during their stay in orbit while filtering the air; 2] ground-based habitat controls (Ground Control, GC; n = 20) placed inside identical Rodent Habitats as FLIGHT, which was placed inside an environmental chamber that can mimic the environmental conditions experienced by the FLIGHT animals over the course of the mission based on recordings over time (e.g., temperature, humidity, and CO_2_ partial pressure), as well as have access to the same NASA Type 12 Nutrient-upgraded Rodent Food Bars, or; 3] standard vivarium controls housed within the animal facility in standard rodent housing (Vivarium, VIV; n = 20). The day prior to launch, the FLIGHT groups were loaded into a transporter, with both groups within their respective cage and isolated with a separator in the transporter (to prevent aggression), and then loaded into the Dragon SpaceX Capsule. Mice were then transferred into Rodent Habitats aboard the ISS, n = 10 per habitat. The day after launch, a set of mice from the same cohort sent from Jackson Labs as the FLIGHT, GC, and VIV groups were sacrificed as a basal control, and tissues were harvested (Baseline; n = 20). Total time aboard the ISS was 34 days. Unbearthing from ISS and splashdown of live mice occurred ~ 35 days after launch, and live mice were collected by the science teams for behavioral and functional testing at Loma Linda Medical Center, immediately followed by euthanization and tissue harvest. A natural disaster (Hurricane Irma) caused dissections of the FLIGHT group (performed Aug-Sept 2017), to differ in time from the two ground controls (May–June, 2018).

### Hind limb unloading and Readaptation to weight-bearing with/without exercise

All experiments were approved by the Wake Forest School of Medicine IACUC. Eighty male C57BL/6 mice were purchased to perform a ground-based analogue (hind limb unloading; HLU) for the RR9 spaceflight experiment. The study was originally powered based on cartilage thinning data presented by Moriyama et al^18^, which measured cartilage thinning in rodents after 4 weeks of immobilization using histological techniques.^.^ Mice arrived at 9 weeks of age, and were housed under a 12:12 h light–dark cycle at 26 °C during a 1 week acclimation period. Ten-week-old, male C57BL/6 mice were then randomly grouped, and either HLU via tail suspension for 30 days (n = 40), or remained weight-bearing (GROUND; n = 40), per our standard protocol. On Day 30, HLU and GROUND mice (n = 10/group) were sacrificed and tissues were collected. Remaining HLU mice were removed from tail suspension to be full weight-bearing, and then all mice (both GROUND and HLU) were enrolled into one of 3 exercise groups (n = 20 per group) from Days 31–80: i] no exercise; ii] climbing exercise performed 3X weekly, with mice running up a custom tube attached to enclosed housing units at both ends (1 m in length X ~ 5 cm diameter), in which the angle was increased bi-weekly from a starting orientation at of 60° from horizontal until the tube was 90°, and investigators ensuring each mouse performed 3 climbs per session, or; iii] running exercise on a treadmill, performed 3X weekly at a speed of 17 cm/s, with 30 min of monitored exercise performed per session. As such, we generated 6 experimental groups in order to examine how a return to weight-bearing with/without exercise could affect joint health after a 30-day reduced weight-bearing period, including GROUND-No exercise; GROUND-Climbing; GROUND-Running; HLU-No exercise; HLU-Climbing; and HLU-Running. The remaining mice were euthanized and their tissues were harvested at Day 80.

HLU procedure: All mice (both HLU and Control) were lightly anesthetized with isoflurane (2% isoflurane, 95% oxygen). Mice enrolled in the HLU groups were both socially housed (n = 2/cage permitting contact) and tail suspended per our standard procedure^16^. Briefly, the HLU animal’s tail was cleaned with 70% alcohol, and treated with Benzoin tincture in order to help with adherence of tape. Traction tape (3 M HealthCare, Two Harbors, MN) was adhered to the tail in a braided manner, and the free ends of the tape were attached to a fish-hook swivel. The swivel was connected to repair clamp, with the barrel treated with Teflon lubricant to help ensure smooth motion across a steel rod running the length of the cage. A 30° angle of the thorax relative to the floor was ensured. Free access to water and nutrition was available to the animals ad libitum.

### Space Shuttle Atlantis (STS-135)

7-week old female C57BL/6 mice (Charles River, Wilmington, MA) were shipped to KSC. At 9 weeks of age, the mice were either grouped as FLIGHT and placed into specialized Animal Enclosure Modules (analogous to Rodent Habitats sent to ISS) to be launched aboard the Space Shuttle Atlantis (STS-135), or served as GROUND controls at KSC, which were housed at similar environmental conditions (temperature, humidity, and carbon dioxide) as in the AEMs on orbit. Mice were skeletally growing at the outset^58^. After 13 days, live mice were returned from orbit, and fresh tissues were harvested within 5 h of landing. The study was part of the NASA Ames Research Center Biospecimen Sharing Program; analyses were performed on tissues collected from n = 15 mice /group. Due to the multiple tissues isolated by one investigator from each limb (e.g., removal and storage of multiple muscles and disarticulation of bones prior to meniscal isolation) during the time-limited process dissection process, in some instances menisci were torn and incomplete. As such, GAG content was only measured from pooled samples involving two whole menisci, involving 8–14 paired samples/group. All procedures were approved by the IACUC at the University of Colorado at Boulder, KSC, NASA Ames research center, and Amgen Corporation.

### Tissue harvest and storage

*RR9 and HLU studies*: The intact right hind limb was carefully removed preserving the synovial joint of the knee and fixed in 10% neutral buffered formalin (n = 10/group). After 3 days, the hind limbs were transferred to 70% ethanol for imaging and histologic analyses. The lateral and medial menisci were extracted from the left hind limb (RR9—n = 20 pairs/group; HLU—n = 7–8 pairs/group; several partial menisci were collected and thus not analyzed) and stored at − 80 °C for both liquid chromatography-mass spectrometry (LC–MS/MS) analysis and dimethylmethylene colorimetric assay to measure sulfated GAGs. *STS-135 study*: Menisci were isolated from the knees of the mice within 3–5 h post-flight to KSC. Menisci were snap frozen in liquid nitrogen, and stored at − 80 °C for GAG analysis.

### Contrast-enhanced microcomputed tomography

The right hind limb was immersed in 1% w/v phosphotungstic acid in 70% ethanol 24-h prior to analysis using microcomputed tomography (microCT80, Scanco Medical AG; Bassersdorf, Switzerland),with isotropic voxels of 10 μm/side, at 70 kV and an intensity of 114 μ A. Knees were scanned while still in phosphotungstic acid. Later, the knees were placed into 70% ethanol for histology.

### Image processing and analysis

The resulting microCT data were imported into Mimics Innovation Suite (v.18.0 × 64) and reoriented to maintain similar relative direction between samples in order to measure the structure of both the articular cartilage and meniscus. Reconstructions and analysis were performed as previously described^16^. For normalization, a rectangular region of 250,000 µm^2^ was sectioned for the articular cartilage lining the medial tibial plateau. Each sample included the spatial location for the tibial-femoral cartilage-cartilage contact point as the reference. This region was defined as the central area of the medial tibial plateau, with minimal coverage from the overlying meniscus^59^. The resulting 3D reconstructed area from this region of interest of the articular cartilage, and the whole medial meniscus, were analyzed. The newly sectioned medial tibial articular cartilage was measured for thickness at 5 locations, including: the femoral-tibial cartilage-cartilage contact point, and at a 250 µm displacement that was lateral, medial, anterior and posterior direction from the contact point. Specifically, we measured the volume of cartilage for the entire medial tibial plateau, as well as the thickness at these named regions for comparison across groups as metrics for degradation. In some instances, contrast enhancing agent infiltration within the intact knee joint was insufficient in order to assure robust measures could be made, and as such those images were excluded.

### Histology and molecular probing

After microCT imaging, the right knees were decalcified in Immunocal (Fisher Scientific, Hampton, NH) and embedded in paraffin for sectioning, as previously described^16^. 5 µm thick coronal sections were analyzed from n = 7–10 samples per group. Slides were stained with hematoxylin and eosin (H&E; Meyer’s hematoxylin & Eosin; Abcam, Cambridge, MA) and safranin-O (0.1% Safranin O; 0.05% Fast Green Solution; SigmaAldrich, St. Louis, MO) staining for semi-quantitative analysis of glycosaminoglycans (GAGs). The Osteoarthritis Research Society International (OARSI) scoring system^60^ was used to evaluate arthritis severity by a blinded expert in murine osteoarthritis. Immunostaining identified expression of matrix metalloproteinase-13 (MMP-13; 1:500; Rabbit Polycolonal IgG; Abcam, Cambridge, MA) presence and ADAM Metallopeptidase with Thrombospondin Type 1 Motif 5 (ADAMTS5; 1:50; Rabbit Polycolonal IgG; Abcam, Cambridge, MA). Quantification of MMP-13 and ADAMTS5 within the articular cartilage and medial meniscus was performed to measure the ratio of peri-cellular positive-to-negative reactivity, throughout the whole tibial plateau and medial meniscus. Histologic evaluation was performed using BIOQUANT OSTEO v.17 (Bioquant Image Analysis Corporation, Nashville, TN). The abundance of these proteins within the articular cartilage and menisci were compared between groups as a metric to characterize an arthritic phenotype.

### Proteomics and pathway analysis

The menisci were processed for proteomic analysis (n = 10 pairs/group), using the techniques and resources of the Proteomics and Metabolomics Shared Resource at the Wake Forest School of Medicine and similar to our previous analysis of mouse femoral head cartilage^16^. Two of the 12 samples (paired menisci) per group collected for analysis were excluded as they were determined to be contaminated with muscle during the dissection process. Briefly, frozen menisci were homogenized in radioimmunoprecipitation (RIPA) buffer containing protease inhibitor and homogenized. Centrifuged supernatant (18,000 g) was subjected to reducing alkylation in the presence of 10 mM dithiothreitol and 30 mM iodoacetamide. The protein concentration was measured after centrifugation (14,000 g) and resuspension in 50 mM ammonium bicarbonate; 5 µg of protein per sample was proteolytically digested using 0.1 µg of sequencing-grade modified trypsin and overnight incubation at 37 °C. The resulting peptides were purified using a C18 desalting spin column, dried and resuspended in water containing 5% (v/v) acetonitrile and 1% (v/v) formic acid for LC–MS/MS analysis.

Samples were analyzed by LC–MS/MS system using a Thermo Orbitrap Velos Pro Mass Spectrometer (Thermo Scientific, Waltham, MA) and a Dionex Ultimate-3000 nano-UPLC system (Thermo Scientific, Waltham, MA). An Acclaim PepMap 100 (C18, 5 μm, 100 Å, 100 μm × 2 cm) trap column and an Acclaim PepMap RSLC (C18, 2 μm, 100 Å, 75 μm × 50 cm) analytical column were employed for peptide separation. Spectra were acquired by data dependent scans consisting of MS/MS scans of the ten most intense ions from the full MS scan with dynamic exclusion option for 30 s enabled. The spectra were searched using Sequest HT algorithm within the Proteome Discoverer v2.2 (Thermo Scientific, Waltham, MA) in combination with the UniProt mouse protein FASTA database (annotated 16,747 entries, December 2015). Label free relative quantification (LFQ) was based on the total peak areas of identified peptides normalized to the total ion current (TIC). Search parameters were as follow: FT-trap instrument, parent mass error tolerance of 10 ppm, fragment mass error tolerance of 0.6 Da (monoisotopic), variable modifications of 16 Da (oxidation) on methionine and fixed modification of 57 Da (carbamidomethylation) on cysteine. Further Ingenuity Pathway Analysis (IPA) mapped the proteins identified by MS analysis to canonical pathways, diseases and biological functions. The canonical pathway outputs were then further examined for interactions within the pathways causing potential diseases. The results were validated with Western blot analysis for associated proteins to further indicate responses characterizing an arthritic phenotype. Western blotting was performed using the following antibodies: p-AKT (Ser 473) 0.5 ug/ml (R&D systems, Inc., Minneapolis, MN), total-AKT 10 ug/ml (Cell Signaling Technology, Danvers, MA), Cleaved Caspase-3 (CC-3) 0.5 ug/ml (R&D systems, Inc., Minneapolis, MN), p70S6 Kinase 0.2 ug/ml (R&D systems, Inc., Minneapolis, MN), and β-actin 0.01 ug/ml (R&D systems, Inc., Minneapolis, MN).

### Quantification of GAG content

The sulfated (s)GAG quantity was measured in the pooled medial and lateral menisci of the left knee from the RR9 groups (n = 8), and from the pooled medial and lateral menisci from individual left and right knees (measured separately) from the STS-135 groups (n = 8–14/group). Menisci were digested with a papain solution containing: 125 ug/ml papain, 0.1 M sodium acetate, 5 mM EDTA, 5 mM L-cysteine-HCl and heated at 60 °C overnight (Sigma-Aldrich). Sulfate GAG content from digests were measured using the dimethylmethylene blue colorimetric assay. A mixture of 180 ul of dimethymethylene blue was added to 20 ul of each sample, with a chondroitin sulfate standard (Sigma-Aldrich, St Louis, MO). Absorbance was set at 525 nm. sGAG content was normalized to total protein from a Bicinchoninic acid assay for RR9 groups (BCA, Sigma-Aldrich, St Louis, MO), or normalized to DNA content for STS-135 groups (PicoGreen double-stranded DNA assay reagent, Invitrogen). GAG contents were compared between groups to characterize an arthritic phenotype. As noted, exclusion of samples was based on availability of intact pairs of menisci isolated during dissections.

### Statistical analysis

Statistics were performed by the investigative team and analyzed using GraphPad Prism 8 (San Diego, CA). To determine effects caused by microgravity, data from the RR9 mission were analyzed using one-way ANOVA comparing flight animals vs GC, VIV, and baseline when examined. Moreover, one-way ANOVA was performed the ground-based microgravity analogue HLU vs GROUND study. Unpaired t-test were used for STS-135 data. Data in text is presented as *p* value for the post hoc test following a significant ANOVA result, or the *t* test; when ANOVAs were appropriate and assumptions of a normal distribution and variance were confirmed were appropriate and assumptions of a normal distribution and variance were confirmed, Bonferroni’s post-hoc tests for multiple comparisons between all groups were performed and results are presented in figures; for all α ≤ 0.05. The statistical analysis utilized with IPA for comparing canonical pathway changes and proteins between groups were calculated using a Right-Tailed Fisher’s Exact Test with α ≤ 0.05.

## Supplementary Information

Supplementary Information.
