# Supplementary material for: Spaceflight and hind limb unloading induces an arthritic phenotype in knee articular cartilage and menisci of rodents
Source: Sci Rep. 2021 May 18;11:10469. doi: 10.1038/s41598-021-90010-2 (PMC8131644; doi:10.1038/s41598-021-90010-2)

SPACEFLIGHT AND HIND LIMB UNLOADING INDUCES AN ARTHRITIC PHENOTYPE IN KNEE ARTICULAR CARTILAGE AND MENISCI OF RODENTS

Andy T. Kwok[1], Nequesha S. Mohamed[2], Johannes F. Plate[2], Raghunatha R. Yammani[3,2], Samuel Rosas[2], Ted A. Bateman[4], Eric Livingston[4], Joseph E. Moore[1], Bethany A. Kerr[5,2], Jingyun Lee[3,6], Cristina M. Furdui[3,6], Li Tan[3], Mary L. Bouxsein[7]; Virginia L. Ferguson[8], Louis S. Stodieck[9], David C. Zawieja[10], Michael D. Delp[11], Xiao W. Mao[12], and Jeffrey S. Willey[1,2]

Departments of [1]Radiation Oncology, [2]Orthopaedic Surgery, [3]Internal Medicine, Section of Molecular Medicine, and [5]Cancer Biology, Wake Forest School of Medicine, Winston-Salem, NC, USA

[4]Department of Biomedical Engineering, University of North Carolina, Chapel Hill; Chapel Hill, NC, USA

[6]Proteomics and Metabolomics Shared Resource, Comprehensive Cancer Center, Wake Forest School of Medicine, Winston-Salem, North Carolina.

[7]Department of Orthopedic Surgery, Beth Israel Deaconess Medical Center, Harvard Medical School; Boston, MA, USA

[8]Department of Mechanical Engineering, University of Colorado at Boulder; Boulder, CO, USA

[9]BioServe Space Technologies, Aerospace Engineering Sciences, University of Colorado at Boulder; Boulder, CO, USA

[10]Department of Medical Physiology, Texas A&M University Medical School; Bryan, TX, USA

[11]Department of Nutrition, Food and Exercise Sciences, Florida State University; Tallahassee, FL, USA

[12]Division of Biomedical Engineering Sciences (BMES), Department of Basic Sciences, Loma Linda University, Loma Linda, CA, USA

Keywords: Rodent Research 9; STS-135; menisci; cartilage; arthritis; spaceflight; tail suspension; microgravity; sedentary; exercise

Address correspondence to:

Jeffrey S. Willey
Department of Radiation Oncology
Wake Forest School of Medicine
Winston-Salem, NC 27157
jwilley@wakehealth.edu
336-713-7637

Supplemental Figure 1

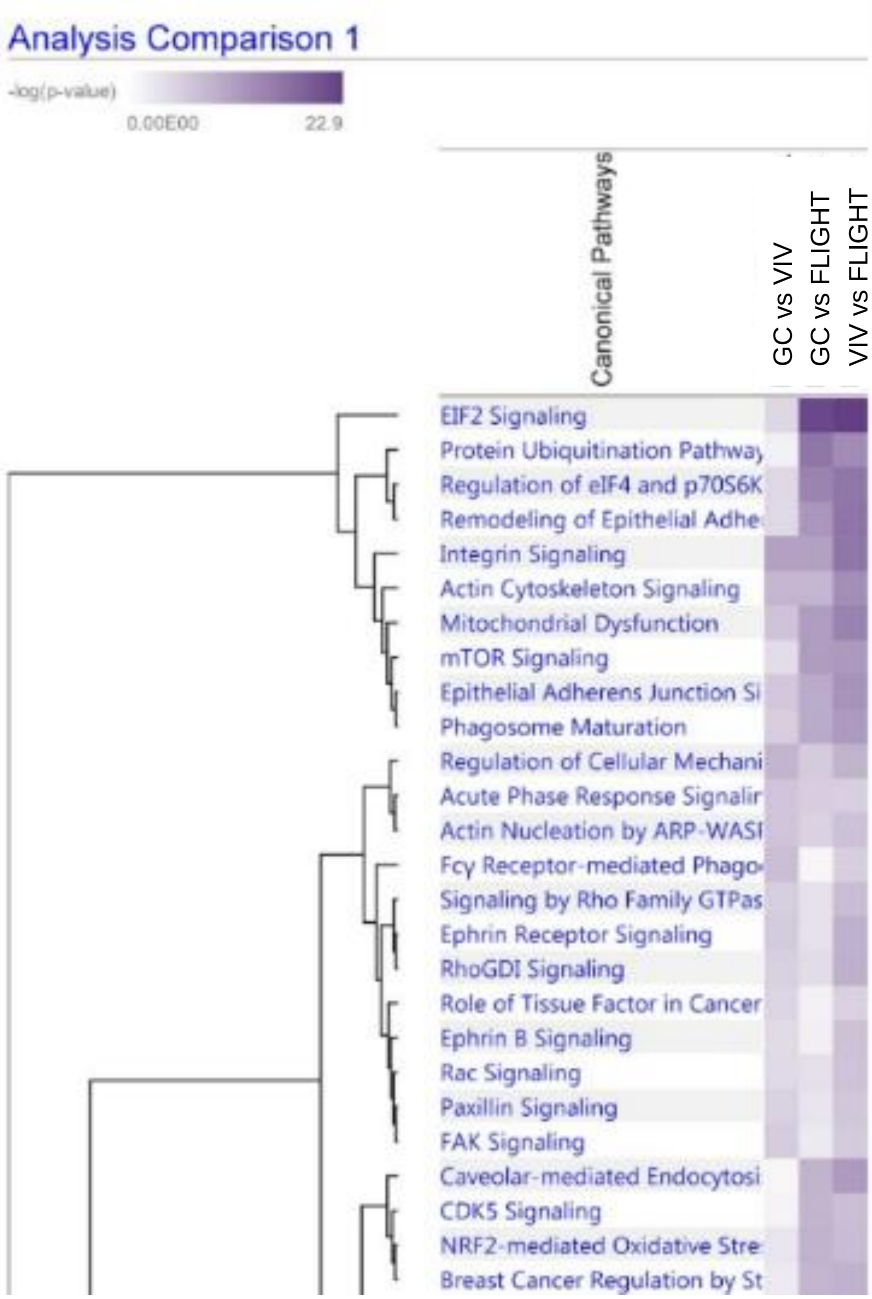

Supplemental Figure 2

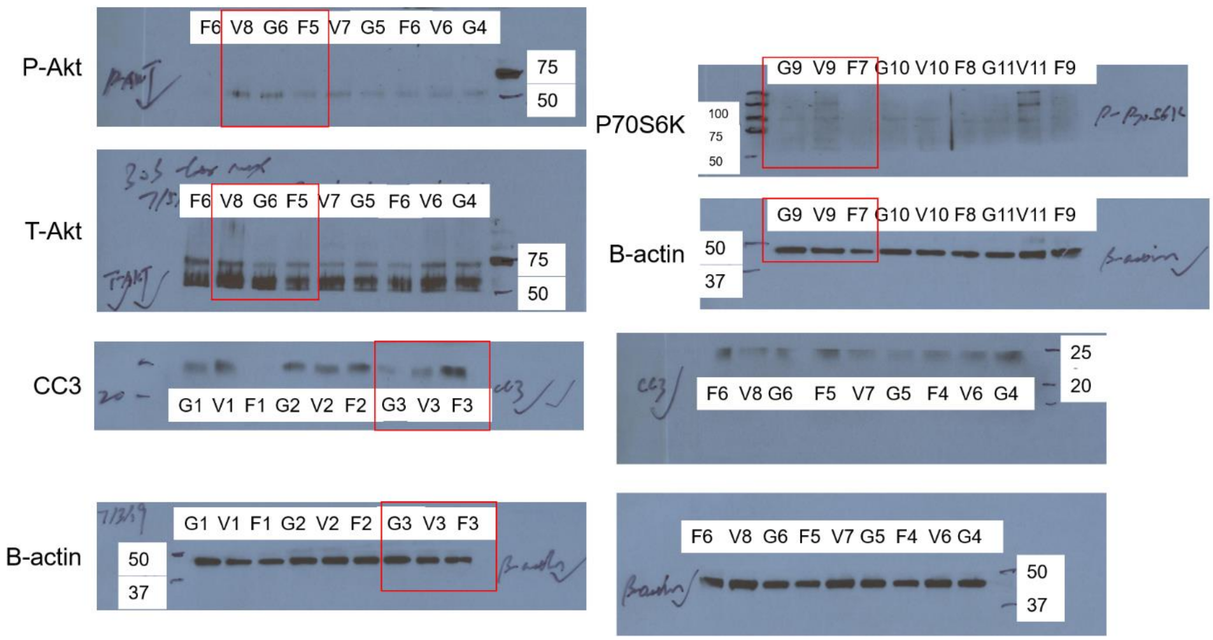

Supplement: Supplementary file 1 — Supplementary Information. [file 41598_2021_90010_MOESM1_ESM.pdf]
